# Performance of WHO-Endorsed Rapid Tests for Detection of Susceptibility to First-Line Drugs in Patients with Pulmonary Tuberculosis in Bangladesh

**DOI:** 10.3390/diagnostics12020410

**Published:** 2022-02-05

**Authors:** S. M. Mazidur Rahman, Md. Fahim Ather, Rumana Nasrin, Mohammad Ariful Hoque, Razia Khatun, Tanjina Rahman, Mohammad Khaja Mafij Uddin, Shahriar Ahmed, Sayera Banu

**Affiliations:** Infectious Diseases Division, International Centre for Diarrhoeal Disease Research, Dhaka 1212, Bangladesh; smmazidur@icddrb.org (S.M.M.R.); fahim.ather@icddrb.org (M.F.A.); rumana.nasrin@icddrb.org (R.N.); ariful.hoque@icddrb.org (M.A.H.); razia@icddrb.org (R.K.); tanjina.rahman@icddrb.org (T.R.); kmuddin@icddrb.org (M.K.M.U.); shahriar.ahmed@icddrb.org (S.A.)

**Keywords:** tuberculosis, drug susceptibility, diagnostic performance, Bangladesh, BACTEC MGIT 960 SIRE, GenoType MTBDR*plus*, Xpert MTB/RIF

## Abstract

The fast and accurate detection of susceptibility in drugs is a major challenge for a successful tuberculosis (TB) control programme. This study evaluated the performance of WHO-endorsed rapid diagnostic tools, such as BACTEC MGIT 960 SIRE (MGIT SIRE), GenoType MTBDR*plus* (MTBDR*plus*) and Xpert MTB/RIF (Xpert), for detecting susceptibility to first-line anti-TB drugs among pulmonary TB patients in Bangladesh. A total of 825 sputum samples with results from drug susceptibility testing (DST) against first-line anti-TB drugs in the MGIT SIRE, MTBDR*plus* and Xpert assays were evaluated and compared with the gold standard proportion susceptibility method of the Lowenstein–Jensen (LJ) medium. The overall sensitivities of MGIT SIRE were 97.6%, 90.0%, 61.3% and 44.9%, while specificities were 89.9%, 94.5%, 91.3% and 92.2% for detection of susceptibility to isoniazid (INH), rifampicin (RIF), streptomycin (STR) and ethambutol (EMB), respectively. For MTBDR*plus*, the sensitivities were 88.0% and 88.7%, and the specificities were 97.4% and 97.8% for the detection of susceptibility to INH and RIF, respectively. Xpert demonstrated a sensitivity and specificity of 94.8% and 99.5%, respectively, for the detection of RIF susceptibility. All tests performed significantly better in retreated TB patients compared with primary TB cases. For detection of RIF and INH susceptibility, all three assays showed almost perfect agreement with the LJ method, although MGIT SIRE exhibited low agreement for STR and EMB. Considering the high performance, shorter turnaround time and ease of use, molecular-based approaches Xpert and MTBDR*plus* can be widely implemented throughout the country for the rapid detection of drug-resistant TB.

## 1. Introduction

Tuberculosis (TB) is an airborne infectious disease caused by *Mycobacterium tuberculosis*, and is the leading cause of death worldwide [1]. According to the World Health Organization (WHO), an estimated 9.9 million people globally developed TB in 2020, of whom about half a million had multidrug-resistant TB (MDR-TB). Bangladesh has a high incidence of TB with 360,000 cases, where an estimated 44,000 people die from the disease annually [1]. The first national survey on drug-resistant TB, which was conducted in 2011, revealed that the prevalence of MDR-TB in Bangladesh was 1.4% in new TB patients and 28.5% in previously treated patients [2]. Failure to promptly diagnose and treat TB, particularly DR-TB, results in increased transmission of the disease along with the development of resistance to additional anti-TB drugs [1,3].

Since the end of 2019, the emergence of SARS-CoV-2 has triggered mortality, morbidity and societal disruption worldwide. In the absence of pharmaceutical interventions initially, many nations resorted to population-wide lockdowns to minimise the spread of the virus and allow their health systems to cope [4]. Concurrently, rapid detection of SARS-CoV-2 virus by real-time RT-PCR [5], RT-LAMP [6,7] and antigen tests [8] were widely adopted to enable case finding and contact tracing. In the year 2020, a provisional data analysis conducted by WHO in 84 countries indicated a 21% shortfall in TB case notifications compared to 2019 due to the COVID-19 pandemic; this was even 28% for the 10 high TB-burden countries. Such reduction in TB diagnosis and care in 2020 could result in an estimated half a million additional TB deaths [9]. However, the rapid diagnostic lesson learned from COVID can enormously drive the TB controlling program through quick detection of the disease as well as a drug resistance pattern that will help improve the TB situation all around the world.

Early detection, with rapid and accurate identification of susceptibility to anti-TB drugs, is crucial for the successful treatment of the disease and control of its transmission. Conventional phenotypic drug susceptibility testing (DST) on the Lowenstein–Jensen (LJ) medium is regarded as the gold standard, and it has been widely used for more than 50 years [10], but using the seed culture, it takes about four to six weeks to obtain the results for drug susceptibility. A liquid culture-based system such as the BACTEC MGIT 960 (MGIT 960) (BD Biosciences, Sparks, NV, USA) is rapid when compared with the solid culture that has been recommended by WHO for TB cultures and DST in low and medium income countries [11]. Cultured isolates obtained from the MGIT 960 method are tested for drug susceptibility using BACTEC MGIT 960 SIRE kits (MGIT SIRE) [12]. The Xpert MTB/RIF (Xpert) assay (Cepheid, Sunnyvale, CA, USA) is an automated semi-nested, real-time quantitative PCR assay that simultaneously detects the *M. tuberculosis* complex and resistance to rifampicin (RIF) [13,14]. The assay also allows for rapid and sensitive detection of pulmonary TB in sputum samples [15,16]. The GenoType MTBDR*plus* (MTBDR*plus*) (Hain Life Science, Nehren, Germany) is a line probe assay with strips containing DNA probes that detect the *M. tuberculosis* complex as well as resistance to RIF and isoniazid (INH). Both the Xpert and MTBDR*plus* assays are performed on sputum specimens as a direct DST, with a turnaround time of a few hours, in contrast to the time required by other culture-based approaches. WHO has endorsed both the Xpert and MTBDR*plus* tests for the quick diagnosis of MDR-TB [17,18]. Despite the availability of many phenotypic and genotypic methods worldwide, the accuracy of a DST varies as it depends on the anti-TB drugs being tested and the methods used [19]. Additionally, the performance of the tests differs depending on the geographical region in which they are conducted [19].

In countries like Bangladesh, which have a high level of MDR-TB, quick and accurate diagnosis of drug resistant TB and MDR-TB is a major challenge for a successful TB control programme. The detection of anti-TB drug susceptibility in Bangladesh is based mainly on the LJ proportion method [2,20]. However, use of the Xpert assay was extended throughout the country recently for the detection of TB and resistance to RIF [21]. The use of MGIT SIRE and MTBDR*plus* tests for the rapid diagnosis of drug-resistant TB has also commenced. Although the diagnostic performance of these phenotypic and molecular techniques for detecting drug susceptibility has been reported from diverse geographical locations, only scanty findings are available from Bangladesh. This study aims to evaluate the performance of WHO-approved rapid diagnostic tools, such as MGIT SIRE, MTBDR*plus* and Xpert, for detecting susceptibility to first-line anti-TB drugs among pulmonary TB patients in Bangladesh.

## 2. Materials and Methods

### 2.1. Study Population

Sputum samples from pulmonary TB patients (both adult and children) were collected as part of a nationwide drug resistance TB surveillance study conducted by the International Centre for Diarrhoeal Disease Research, Bangladesh (icddr,b) from 2011 to 2015. The survey included 14 hospitals from all geographical divisions in Bangladesh [20]. In this sentinel surveillance study, newly registered smear-positive pulmonary TB patients were enrolled through a systematic sampling strategy. All presumptive pulmonary TB patients were screened for the presence of specific symptoms such as persistent cough for more than three weeks, a daily rise in temperature in the evenings (>38 °C) for more than two weeks, and abnormal chest radiographic findings (TB may be present with any of these three findings but the chance of TB is high with the combination). In addition, other clinical parameters such as chest pains, fatigue, loss of appetite, weight loss and night sweats were also assessed [22]. Both primary (new TB cases) and retreated (previously treated patients with treatment failure, relapse or loss to follow-up) TB cases were included in the study. Patients who had already started anti-TB therapy at the time of diagnosis were ineligible to participate. The research and ethical review committees of the icddr,b approved the study. Only patients who consented to provide written informed permission to participate in the study were included.

### 2.2. Specimen Processing and Culture

All sputum specimens were decontaminated and processed by following Petroff’s NaOH method [23]. Briefly, an equal volume of N-acetyl-L-cysteine (NALC)-NaOH-Na-citrate solution (0.5% NALC, 4% NaOH and 2.94% Na-citrate) was added to the sputum specimen in a 50 mL centrifuge tube and incubated for 20 min at room temperature. The tube was then filled with sterile phosphate buffer saline (PBS) (pH 6.8) up to the 40 mL mark, vortexed well and centrifuged at 3000× *g* for 20 min. The supernatant was decanted carefully, and the resultant sediment was resuspended in 1.0 mL of PBS. Two loops of processed sputum were inoculated on two LJ slants and 500 µL in an MGIT 960 tube with a PANTA supplement. PANTA is a cocktail of antibiotics comprising polymyxin-B, amphotericin-B, nalidixic acid, trimethoprim and azlocillin that is used to kill other bacteria to promote mycobacterial growth [24]. Cultures were incubated at 37 °C for up to eight weeks for LJ slants and 42 days for the MGIT 960 system. LJ slants were checked once per week and MGIT cultures were monitored continuously through the automated MGIT 960 instrument.

### 2.3. Drug Susceptibility Testing

Cultured isolates obtained from processed sputum samples were subjected to susceptibility testing using the LJ proportion method and MGIT SIRE. The Xpert and MTBDR*plus* assays were performed on direct and processed sputum, respectively, by following the manufacturer’s instructions.

#### 2.3.1. LJ Proportion Method

The DST of cultured *M. tuberculosis* isolates was performed by following the standard LJ proportion method, as described previously [25]. LJ media containing drugs were prepared using streptomycin (STR) at 4.0 µg/mL, INH at 0.2 µg/mL, RIF at 40.0 µg/mL and ethambutol (EMB) at 2.0 µg/mL. Stock solutions of each drug were prepared from a reference powder, and all the media were prepared in-house to maintain their sterility and performance. A 1.0 McFarland standard suspension of *M. tuberculosis* isolate was prepared in sterile distilled water using freshly grown cultured colonies. The suspension was then serially diluted 10-fold in sterile distilled water from 10^1^ to 10^4^, inoculated onto LJ slants, with or without drugs, using a platinum loop with an internal diameter of 3.0 mm calibrated to 10 µL. LJ media were incubated at 37 °C, and results were read at 28, 35 and 42 days, depending on the growth of the control media. An isolate was considered as ‘resistant’ when the number of colonies grown on the drug-containing media was at a level equal to or 1% above the drug-free media.

#### 2.3.2. BACTEC MGIT 960 SIRE

DST on the MGIT 960 system was performed by following the manufacturer’s instructions with SIRE kits containing STR at 1.0 µg/mL, INH at 0.1 µg/mL, RIF at 1.0 µg/mL and EMB at 5.0 µg/mL [26]. A suspension of *M. tuberculosis* was prepared from a culture-positive MGIT tube that had been detected by MGIT. In each 7 mL MGIT tube, 800 µL of an MGIT SIRE supplement was added, followed by 100 µL of a drug and a 500 µL suspension of test isolate. A growth control tube with a SIRE supplement without drugs was included for each isolate. All the tubes were inserted in the MGIT 960 system, and the susceptibility results were reported automatically by the system after comparing the relative growth ratio between the drug-containing and drug-free growth control tubes. Detailed information on the BACTEC MGIT instrument and susceptibility testing reagents with images can be found on the manufacturer’s website (https://www.bd.com/en-us/offerings/capabilities/microbiology-solutions/mycobacteria-testing/bd-bactec-mgit-automated-mycobacterial-detection-system, accessed on 5 December 2021).

#### 2.3.3. GenoType MTBDR*plus* Assay

The MTBDR*plus* assay is based on DNA-strip technology, which permits the detection of the *M. tuberculosis* complex as well as susceptibility to RIF and INH. The test was performed on decontaminated and concentrated sputum samples by following the manufacturer’s instructions [27]. DNA was extracted from the concentrated sputum sample, amplified by PCR, and the PCR product was then hybridised to specific oligo-nucleotide probes immobilised on the strip. The strip contains six control zones for verification of the test procedures. The control zones include a conjugate control zone (CC), an amplification control zone (AC), an *M. tuberculosis* complex specific control zone (TUB) and three locus control zones (rpoB for RIF, katG and inhA for INH). The result was considered valid if all the control zones appeared on the strip. In case of heteroresistant results—strips that showed positive bands for both mutation probes and corresponding wild-type probes—the samples were categorised as ‘resistant’. Additional information about the MTBDR*plus* assay, including the instruments and reagents, can be obtained on the manufacturer’s website—Hain Lifescience (https://www.hain-lifescience.de/en/products/microbiology/mycobacteria/tuberculosis/genotype-mtbdrplus.html, accessed on 5 December 2021).

#### 2.3.4. Xpert MTB/RIF Assay

The Xpert assay was performed directly on unprocessed sputum samples by following the manufacturer’s instructions [28]. The Xpert sample reagent was added to the sputum sample at a ratio of 2:1 in a 15 mL falcon tube. The tube was then incubated for 15 min at room temperature, mixed well by inverting the tubes twice during this incubation period, and finally 2 mL was transferred to an Xpert cartridge (Version 3.0) and loaded into the module of the GeneXpert IV machine. The results for detection of *M. tuberculosis* and RIF susceptibility were obtained within two hours of the completion of the test. The manufacturer’s website has detailed information on the Xpert assay device and cartridge (https://www.cepheid.com/en/tests/Critical-Infectious-Diseases/Xpert-MTB-RIF, accessed on 5 December 2021).

### 2.4. Quality Control

For the purpose of quality control, a susceptible strain (H37Rv, from American Type Culture Collection, ATCC) and our laboratory-defined resistant strain (SB256) were tested by all DST methods. H37Rv and SB256 are known strains susceptible and resistant to STR, INH, RIF and EMB, respectively [29]. Both the LJ proportion and MGIT SIRE methods detected all four first-line drugs as sensitive to H37Rv and resistant to SB256 strains, while MTBDR*plus* detected RIF and INH, and Xpert detected RIF as sensitive to H37Rv and resistant to SB256.

### 2.5. Statistical Analysis

Demographic and clinical data, and laboratory findings were entered and analysed by the SPSS version 15.0 software package (Statistical Package for the Social Sciences Inc, Chicago, IL, USA). A chi-square test or Fisher exact probability test was used to examine the association of drug susceptibility patterns between the primary and retreated groups of TB patients. The sensitivity, specificity, positive predictive value (PPV) and negative predictive value (NPV) of MGIT SIRE, MTBDR*plus* and Xpert tests for the detection of STR, INH, RIF and EMB susceptibility were determined by a comparison with the gold standard LJ proportion method. The diagnostic yield of the methods was compared between the primary and retreated groups. A comparison of proportion was carried out by a chi-square test, with *p* < 0.05 regarded as evidence of a statistically significant difference. Agreement between the methods was assessed by using Cohen’s kappa statistic. The kappa value was interpreted as <0.2, slight; 0.21–0.4, fair; 0.41–0.6, moderate; 0.61–0.8, substantial; and ≥0.81–1.00, almost perfect or in perfect agreement [30].

## 3. Results

### 3.1. Demographic and Clinical Characteristics of TB Patients

The current study comprised 825 TB patients with DST results available for all four methods—the LJ proportion technique, MGIT SIRE, MTBDR*plus* and Xpert. Among the TB patients, 68% (*n* = 561) were male and the remainder female. The median age of the patients was 30, with an interquartile range of 22 to 45 years, while 51% of the patients were between the ages of 21 and 40 (Table 1). Approximately 16% of the patients had previous exposure to TB patients, whereas about 32% of the patients had a previous history of TB and treatment with anti-TB drugs. Around 62% of the patients were from urban areas. Most of the patients (77.3%) were from the Chittagong, Dhaka and Rajshahi divisions (Table 1).

### 3.2. Drug Resistance Pattern of Isolates Based on the LJ Proportion Method

Susceptibility testing against INH, RIF, STR and EMB by the LJ proportion method revealed that 483 (58.5%) of the isolates were sensitive, and 159 (19.3%) were resistant to all four first-line anti-TB drugs (Table 2). A total of 225 (27.3%) isolates were detected as MDR-TB by the LJ proportion method. Mono resistance to STR, INH, RIF and EMB was identified in 84 (10.2%), six (0.7%), three (0.4%) and one (0.1%) of the isolates, respectively. STR mono resistance was significantly higher among primary TB cases (*p* < 0.0001) than retreated cases. The number of isolates sensitive to all four drugs was significantly higher in primary TB patients compared with the retreated group (*p* < 0.0001). In contrast, the number of isolates with MDR-TB or resistant to all four first-line anti-TB drugs was significantly higher in the retreated group (*p* < 0.0001) (Table 2).

### 3.3. Performance of the MGIT SIRE Assay

Table 3 represents the overall performance of the MGIT SIRE assay and its performance in the primary and retreated groups for the detection of susceptibility to four first-line anti-TB drugs. Compared with the LJ proportion method, the overall sensitivities of the MGIT SIRE for the detection of INH, RIF, STR and EMB susceptibility were 97.6% (95% CI, 94.9–99.1), 90.0% (95% CI, 85.4–93.6), 61.3% (95% CI, 55.4–67.0) and 44.9% (95% CI, 37.7–52.4), respectively. The sensitivity of the assay was also compared between the primary and retreated groups, and it was evident that the sensitivity of MGIT SIRE was much higher in the retreated cases for all four drugs. The sensitivities for detection of INH susceptibility in the primary versus the retreated group were 93.6% (95% CI, 82.5–98.7) vs. 98.5% (95% CI, 95.7–99.7) (*p* < 0.005); for RIF they were 79.3% (95% CI, 60.3–92.0) vs. 91.5% (95% CI, 86.8–95.0) (*p* < 0.0001); for STR they were 42.9% (95% CI, 33.6–52.6) vs. 73.1% (95% CI, 65.9–79.6) (*p* < 0.0001); and for EMB they were 36.4% (95% CI, 17.2–59.3) vs. 46.1% (95% CI, 38.3–54.0) (*p* < 0.01) (Table 3). Even the sensitivities for detecting STR and EMB susceptibility were higher in the retreated group when compared with the assay’s overall sensitivities. The assay’s overall specificities for INH, RIF, STR and EMB susceptibility were 89.9% (95% CI, 87.2–92.3), 94.5% (95% CI, 92.3–96.2), 91.3% (95% CI, 88.6–93.5) and 92.2% (95% CI, 89.8–94.1), respectively. There were no significant variations in specificities for detection of the four first-line anti-TB drugs between the primary and retreated groups. The specificities for detecting INH susceptibility in the primary versus the retreated groups were 90.0% (95% CI, 87.1–92.5) vs. 89.1% (95% CI, 78.8–95.5); for RIF they were 94.5% (95% CI, 92.2–96.3) vs. 93.9% (95% CI, 85.2–98.3); for STR they were 91.7% (95% CI, 88.8–94.1) vs. 89.1% (95% CI, 80.9–94.7); and for EMB they were 92.0% (95% CI, 89.4–94.1) vs. 93.1% (95% CI, 86.4–97.2) (Table 3). The MGIT SIRE had an ‘almost perfect’ agreement with the LJ proportion method for detecting both INH and RIF susceptibility (*k* value = 0.83), but moderate agreement for STR (*k* value = 0.55) and EMB (*k* value = 0.41) (Table 3).

### 3.4. Performance of the MTBDRplus Assay

The overall sensitivity and specificity of the MTBDR*plus* assay for the detection of susceptibility to INH were 88.0% (95% CI, 83.3–91.8) and 97.4% (95% CI, 95.7–98.5), and that for the RIF were 88.7% (95% CI, 83.9–92.5) and 97.8% (95% CI, 96.3–98.8), respectively. The sensitivity of the assay was significantly higher in the retreated cases for both RIF and INH. The sensitivities in primary versus retreated TB patients were 80.9% (95% CI, 66.7–90.9) vs. 89.7% (95% CI, 84.6–93.5) (*p* < 0.005) for INH, and 75.9% (95% CI, 56.5–89.7) vs. 90.6% (95% CI, 85.6–94.2) (*p* < 0.0001) for RIF, respectively. The specificities were also higher in the retreated group than primary for detecting susceptibility to RIF (100%; 95% CI, 94.6–100 vs. 97.5%; 95% CI, 95.8–98.7) (*p* < 0.01) and INH (98.4%; 95% CI, 91.6–100 vs. 97.3%; 95% CI, 95.5–98.5) (*p* = 0.328) (Table 4). The MTBDR*plus* exhibited ‘almost perfect’ agreement with the LJ proportion method for the detection of both INH (*k* value = 0.87) and RIF (*k* value = 0.88) susceptibility (Table 4).

### 3.5. Performance of the Xpert MTB/RIF Assay

For the Xpert assay, the overall sensitivity and specificity for the detection of susceptibility to RIF were 94.8% (95% CI, 91.1–97.3) and 99.5% (95% CI, 98.5–99.9), respectively. The sensitivity was significantly higher in the retreated group (96.0%; 95% CI, 92.3–98.3) compared with the primary group (86.2%; 95% CI, 68.3–96.1) (*p* < 0.0001). However, the specificity was almost the same for both groups (100%; 95% CI, 94.6–100 vs. 99.4; 95% CI, 98.4–99.9% in retreated vs. primary TB patients). The assay exhibited ‘almost perfect’ agreement with the LJ proportion method for the detection of RIF susceptibility (*k* = 0.95) (Table 5).

## 4. Discussion

Making a rapid and accurate diagnosis of TB and assessing its susceptibility to anti-TB drugs are crucial for effective treatment of the disease, patient management and infection control. Furthermore, TB with drug-resistant strains is considerably more difficult to treat than TB that is susceptible to drugs [3]. The rising incidence of drug-resistant TB throughout the world has highlighted the necessity for accurate and speedy detection of drug resistance to initiate early and proper treatment of patients [31]. Several diagnostic methods are available for quick and reliable diagnosis of drug-resistant TB, but there is little information on their performance in Bangladesh. Therefore, this study aimed to assess the performance of WHO-approved rapid diagnostic tools in the detection of susceptibility to first-line anti-TB drugs among pulmonary TB patients in Bangladesh.

This study found that the MGIT SIRE had an overall good performance, with sensitivity and specificity of more than 90%, and almost perfect agreement (*k* = 0.83) with the LJ proportion method for detecting susceptibility to both INH and RIF. The findings were in agreement with previous studies, as demonstrated by a meta-analysis where the pooled sensitivities and specificities of the MGIT SIRE were more than 90% for detection of INH and RIF susceptibility [19,32,33]. On the other hand, the assay’s performance in detecting susceptibility to EMB and STR was poor, with <60% sensitivity and a moderate agreement (*k* < 0.6) compared with the LJ proportion method. However, the specificity was >90% for both EMB and STR. This revealed that the MGIT SIRE assay could detect the EMB and STR susceptible isolates more accurately than resistant ones. Overall low performance has been reported for EMB and STR in multiple studies across different geographical locations since the scale-up of the platform [19,34]. These findings suggest a variable performance of the MGIT SIRE across different countries and laboratories for the detection of susceptibility to STR and EMB. Recent studies conducted in China and India demonstrated high performance of MGIT SIRE in the detection of EMB and STR susceptibility, with sensitivity at around 90.0% and specificity at around 95% [33,35]. In South Africa, compared with the agar proportion method, the sensitivity of MGIT SIRE for both drugs was satisfactory (around 90%) but specificity was below 40%. In our previous study, we investigated the discordance across several DST methods, including MGIT SIRE, for detection of susceptibility of four first-line anti-TB drugs, where high concordance across the methods was found for INH and RIF, but low concordance for EMB and STR [36].

According to the findings, the molecular approaches were consistent with the overall DST results of the LJ proportion method. Both the MTBDR*plus* and Xpert assays showed almost perfect agreement with the proportion method in the identification of RIF-resistant cases (*k* = 0.88 and 0.95, respectively), with sensitivities of 88.7% and 94.8%, respectively, and specificities of 97.8% and 99.5%, respectively. The investigation revealed that like the MGIT SIRE, the molecular methods identified RIF-susceptible isolates more accurately than resistant ones. Similar findings were also observed in a multi-centre evaluation of the MTBDR*plus* assay against the gold standard proportion method in China, where the sensitivity and specificity for RIF resistance detection were 88.33% and 97.66%, respectively [37]. On the other hand, a multi-centre implementation study by Boehme et al. detected RIF-resistant cases by the Xpert assay, with the sensitivity and specificity of 94.4% and 98.3%, respectively [38]. However, in terms of detecting INH susceptibility with the MTBDR*plus* assay, the sensitivity and specificity were 88.0% and 97.4%, respectively, compared with the conventional DST. The results are consistent with other studies, which reported the sensitivity of MTBDR*plus* in detecting INH susceptibility between 72% and 92%, and specificity between 93% and 100% [39,40,41]. The reduced sensitivity of MTBDR*plus* in detecting INH and RIF-resistant isolates (88.0% and 88.7%, respectively) might be attributed to the inability of the test to detect mutations in genes other than regions targeted by the assay [41]. Future research should be conducted to investigate in depth the mutational profiles of such isolates in different geographical locations, which will assist manufacturers in enhancing the accuracy of the MTBDR*plus* assay in detecting RIF and INH-resistant isolates.

This research also investigated the performance of the assays between the primary and retreated group of TB patients for detecting susceptibility to first-line anti-TB drugs. All the assays performed significantly better in identifying resistant cases among retreated TB patients than primary patients. For example, in the case of determining RIF resistant cases, the sensitivities of MGIT SIRE, MTBDR*plus* and Xpert assay for retreated vs. primary TB patients were 91.5, 90.6, 96.0% vs. 79.3, 75.9, 86.2% respectively. However, the reason for increased sensitivities of the assays among retreated TB cases compared to the primary TB patients is unknown. Future studies should be performed to investigate the mechanisms behind such disparities. It has been shown that the rate of drug-resistant TB and MDR-TB is higher among the retreated group of TB patients worldwide, including Bangladesh [1,20,42]. Therefore, the application of these assays would be beneficial for rapid identification of drug susceptibility among TB patients who endure treatment failure or relapse. This would eventually guide clinicians to begin anti-TB medication of patients as early as possible with appropriate regimens.

From an operational standpoint, the widespread deployment of Xpert and MTBDR*plus* tests would be more practical owing to shorter turnaround times, fewer repeats and ease of handling over the culture-based MGIT 960 SIRE, as demonstrated in our earlier investigations [36]. As the Xpert and MTBDR*plus* tests are based on sputum samples, results can be acquired within three and eight hours, respectively. MGIT SIRE, on the other hand, being a culture-based approach, requires an average of 11 days to obtain DST results, with an initial four to six weeks for sufficient culture growth of the isolates. Furthermore, owing to contamination or inadequate growth, MGIT SIRE requires more repeat testing than the others [36]. Although the Xpert assay can only identify RIF resistance, the benefit is that RIF resistance is considered to be a surrogate marker of MDR-TB because the majority of RIF-resistant individuals are also INH-resistant [43]. With the current evidence of good performance, there is optimism that the Xpert assay could play a crucial role in the control of drug-resistant TB in Bangladesh because it has been greatly expanded throughout the country [21]. While MTBDR*plus* can detect both RIF and INH susceptibility, its performance in detecting the resistant isolates for both these drugs was lower than the MGIT SIRE, and even lower than the Xpert assay for detecting RIF-resistant isolates. Future studies to explore in detail the mutational profiling of such false susceptible isolates would provide significant information to the manufacturer to enhance the assay’s performance in places like Bangladesh.

There were a few limitations to this investigation. In this study, only smear-positive pulmonary TB patients were included. Therefore, the performance of the assays in the detection of anti-TB drug susceptibility among smear-negative pulmonary TB patients could not be determined. This study did not assess the impact of determining anti-TB drug susceptibility on the treatment outcomes of TB patients since a long-term follow-up of the patients in the study was not conducted. Furthermore, no additional sequencing was performed on isolates that exhibited discordant susceptibility results in different assays. However, the findings of this research show that WHO-approved rapid tests are effective in identifying the susceptibility of most potent first-line anti-TB drugs, such as RIF and INH, in pulmonary TB patients in Bangladesh.

## 5. Conclusions

In conclusion, all the WHO-approved rapid methods—Xpert, MTBDR*plus* and MGIT SIRE—demonstrated good agreement with the gold standard LJ proportion method for detecting RIF and INH susceptibility. However, the performance of MGIT SIRE was found to be poor in detecting STR and EMB susceptibility. All the assays performed better in detecting anti-TB drug susceptibility among the retreated group of TB patients compared with the primary group. Considering the excellent performance, shorter turnaround time and ease of use, the molecular-based approaches—Xpert and MTBDR*plus*—can be extensively deployed throughout the country for rapid detection of drug-resistant TB.

## Figures and Tables

**Table 1:** Demographic and clinical characteristics of 825 pulmonary TB patients.

Variable	Label	Number of Patients (*n* = 825)	Frequency (%)
Sex	Male	561	68.0
	Female	264	32.0
Age (Years)	≤20	158	19.2
	21–40	421	51.0
	41–60	199	24.1
	>60	47	5.7
Smoking	Yes	325	39.4
	No	500	60.6
Drug user	Yes	45	5.5
	No	780	94.5
Dwelling	Rural	317	38.4
	Urban	508	61.6
Exposure to TB patients	Yes	130	15.8
	No	695	84.2
Previous history of TB	Yes	267	32.4
	No	558	67.6
Geographic	Chittagong	239	29.0
	Dhaka	235	28.5
	Rajshahi	163	19.8
	Mymensingh	72	8.7
	Khulna	42	5.1
	Sylhet	37	4.5
	Barishal	37	4.5

**Table 2:** Susceptibility results of INH, RIF, STR and EMB among pulmonary TB patients as determined by the LJ proportion method.

Susceptibility Pattern	Total Cases (*n* = 825) *n* (%)	Primary Cases (*n* = 558) *n* (%)	Retreated Cases (*n* = 267) *n* (%)	*p* Value
All sensitive	483 (58.5)	431 (77.2)	52 (19.5)	<0.0001
All resistant	159 (19.3)	15 (2.7)	144 (53.9)	<0.0001
Only STR ^R^	84 (10.2)	74 (13.3)	10 (3.7)	<0.0001
Only INH ^R,^*	6 (0.7)	5 (0.9)	1 (0.4)	0.670
Only RIF ^R,^*	3 (0.4)	2 (0.4)	1 (0.4)	
Only EMB ^R,^*	1 (0.1)	1 (0.2)	0 (0)	
INH ^R^ + RIF ^R^	21 (2.5)	3 (0.5)	18 (6.7)	<0.0001
INH ^R^ + RIF ^R^ + STR ^R^	21 (2.5)	3 (0.5)	18 (6.70	<0.0001
INH ^R^ + RIF ^R^ + EMB ^R^	24 (2.9)	4 (0.7)	20 (7.5)	<0.0001
STR ^R^ + INH ^R^	18 (2.2)	16 (2.9)	2 (0.7)	0.072
STR ^R^ + RIF ^R,^*	2 (0.2)	2 (0.4)	0 (0)	0.562
STR ^R^ + EMB ^R,^*	2 (0.2)	1 (0.2)	1 (0.4)	
STR ^R^ + INH ^R^ + EMB ^R,^*	1 (0.1)	1 (0.2)	0 (0)	

* Fisher exact probability test; INH ^R^, isoniazid resistant; RIF ^R^, rifampicin resistant; STR ^R^, streptomycin resistant; EMB ^R^, ethambutol resistant.

**Table 3:** Performance of MGIT 960 SIRE assay for detection of isoniazid, rifampicin, streptomycin, and ethambutol susceptibility.

Methods	Patient Group	Drugs	Susceptibility	LJ Proportion Method		Sensitivity % (95% CI)	Specificity % (95% CI)	PPV % (95% CI)	NPV (95% CI)	Level of Agreement (*k*-Value)
				R	S					
MGIT 960 SIRE assay	Overall (*n* = 825)	INH	R	244	58	97.6	89.9	80.8	98.9	0.83
			S	06	517	(94.9–99.1)	(87.2–92.3)	(76.7–84.3)	(97.5–99.5)	
		RIF	R	207	33	90.0	94.5	86.3	96.1	0.83
			S	23	562	(85.4–93.6)	(92.3–96.2)	(81.8–89.8)	(94.3–97.3)	
		STR	R	176	47	61.3	91.3	78.9	81.6	0.55
			S	111	491	(55.4–67.0)	(88.6–93.5)	(73.7–83.3)	(79.2–83.7)	
		EMB	R	84	50	44.9	92.2	62.7	85.1	0.41
			S	103	588	(37.7–52.4)	(89.8–94.1)	(55.–69.6)	(83.4–86.7)	
	Primary cases (*n* = 558)	INH	R	44	51	93.6	90.0	46.3	99.4	0.57
			S	03	460	(82.5–98.7)	(87.1–92.5)	(39.7–51.1)	(98.1–9.8) *	
		RIF	R	23	29	79.3	94.5	44.2	98.8	0.54
			S	6	500	(60.3–92.0)	(92.2–96.3)	(34.7–54.2)	(97.6–9.4) *	
		STR	R	48	37	42.9	91.7	56.8	86.5	0.38
			S	64	409	(33.6–52.6)	(88.8–94.1)	(47.1–65.4)	(84.5–8.3) *	
		EMB	R	8	43	36.4	92.0	15.7	97.3	0.17
			S	14	493	(17.2–59.3)	(89.4–94.1)	(9.1–25.8)	(96.3–8.0) *	
	Retreated cases (*n* = 267)	INH	R	200	7	98.5	89.1	96.6	95.0	0.90
			S	03	57	(95.7–99.7) *	(78.8–95.5)	(93.4–98.3) *	(86.0–98.3)	
		RIF	R	184	4	91.5	93.9	97.9	78.5	0.80
			S	17	62	(86.8–95.0) *	(85.2–98.3)	(94.7–99.2) *	(69.7–85.2)	
		STR	R	128	10	73.1	89.1	92.8	63.6	0.57
			S	47	82	(65.9–79.6) *	(80.9–94.7)	(87.6–95.9) *	(57.5–69.2)	
		EMB	R	76	7	46.1	93.1	91.6	51.6	0.34
			S	89	95	(38.3–54.0) *	(86.4–97.2)	(83.9–95.8) *	(47.9–55.4)	

* Significant differences of proportion between the primary and retreated group of TB patients; INH, isoniazid; RIF, rifampicin; STR, streptomycin; EMB, ethambutol; R, resistant; S, sensitive.

**Table 4:** Performance of MTBDR*plus* assay for detection of rifampicin and isoniazid susceptibility.

Methods	Patient Group	Drugs	Susceptibility	LJ Proportion Method		Sensitivity % (95% CI)	Specificity % (95% CI)	PPV % (95% CI)	NPV (95% CI)	Level of Agreement (*k*-Value)
				R	S					
Line Probe Assay	Overall	INH	R	220	15	88.0	97.4	93.6	94.9	0.87
			S	30	560	(83.3–91.8)	(95.7–98.5)	(89.9–96.0)	(93.0–96.3)	
	(*n* = 825)	RIF	R	204	13	88.7	97.8	94.0	95.7	0.88
			S	26	582	(83.9–92.5)	(96.3–98.8)	(90.2–96.4)	(94.0–97.0)	
	Primary cases	INH	R	38	14	80.9	97.3	73.1	98.2	0.75
			S	9	497	(66.7–90.9)	(95.5–98.5)	(61.4–82.3)	(96.8–99.0) *	
	(*n* = 558)	RIF	R	22	13	75.9	97.5	62.9	98.7	0.67
			S	7	516	(56.5–89.7)	(95.8–98.7)	(48.8–75.0)	(97.5–99.3) *	
	Retreated cases	INH	R	182	1	89.7	98.4	99.5	75.0	0.80
			S	21	63	(84.6–93.5) *	(91.6–100)	(96.3–99.9) *	(66.7–81.8)	
	(*n* = 267)	RIF	R	182	0	90.6	100	100 *	77.7	0.83
			S	19	66	(85.6–94.2) *	(94.6–100)		(69.4–84.2)	

* Significant differences of proportion between the primary and retreated group of TB patients; INH, isoniazid; RIF, rifampicin; R, resistant; S, sensitive.

**Table 5:** Performance of Xpert MTB/RIF assay for detection of rifampicin susceptibility.

Methods	Patient Group	Drugs	Susceptibility	LJ Proportion Method		Sensitivity % (95% CI)	Specificity % (95% CI)	PPV % (95% CI)	NPV (95% CI)	Level of Agreement (*k*-Value)
				R	S					
Xpert MTB/RIF assay	Overall (*n* = 825)	RIF	R	218	03	94.8	99.5	98.6	98.0	0.95
			S	12	592	(91.1–97.3)	(98.5–99.9)	(95.9–99.6)	(96.6–98.9)	
	Primary cases	RIF	R	25	3	86.2	99.4	89.3	99.3	0.87
			S	4	526	(68.3–96.1)	(98.4–99.9)	(72.8–96.3)	(98.2–99.7) *	
	Retreated cases	RIF	R	193	0	96.0	100	100 *	89.2	0.92
			S	19	66	(92.3–98.3) *	(94.6–100)		(80.7–94.2)	

* Significant differences of proportion between the primary and retreated group of TB patients; RIF, rifampicin; R, resistant; S, sensitive.

## Data Availability

The data presented in this study are available on request from the corresponding author. The data are not publicly available due to ethical restrictions.
